# Supplementary material for: Alpha-Lipoic Acid Treatment Reduces the Levels of Advanced End Glycation Products in Type 2 Diabetes Patients with Neuropathy
Source: Biomedicines. 2025 Feb 11;13(2):438. doi: 10.3390/biomedicines13020438 (PMC11852413; doi:10.3390/biomedicines13020438)
Supplement: Supplementary file 1 [file biomedicines-13-00438-s001.zip › biomedicines-3452508-supplementary.pdf]

Supplementary Table S1. Correlations of AGE, sRAGE and AGE/sRAGE ratio with anthropometric and routine laboratory parameters in neuropathic patients before and after ALA treatment

	**AGE (AU/µg protein)**			
	**Before ALA**		**After ALA**	
	**r**	***p***	**r**	***p***
Age (years)	0.20	0.142	0.19	0.191
BMI (kg/m^2)	−0.01	0.937	−0.13	0.357
Glucose (mmol/L)	−0.13	0.345	−0.09	0.549
HbA1C (%)	−0.04	0.790	−0.10	0.502
Creatinine (µmol/L)	0.23	0.107	0.24	0.102
Uric Acid (µmol/L)	0.09	0.519	0.16	0.298
Total cholesterol (mmol/L)	0.07	0.617	0.16	0.284
HDL-C (mmol/L)	0.22	0.112	0.37	0.093
LDL-C (mmol/L)	0.05	0.724	0.08	0.608
Non-HDL-C (mmol/L)	−0.02	0.881	0.03	0.844
hsCRP (mg/L)	−0.20	0.166	−0.02	0.888
	**sRAGE (pg/mL)**			
	**Before ALA**		**After ALA**	
	**r**	***p***	**r**	***p***
Age (years)	0.03	0.841	0.14	0.314
BMI (kg/m^2)	−0.05	0.671	0.07	0.612
Glucose (mmol/L)	0.09	0.532	−0.22	0.121
HbA1C (%)	0.09	0.507	0.126	0.395
Creatinine (µmol/L)	0.16	0.248	0.11	0.447
Uric Acid (µmol/L)	−0.04	0.754	−0.07	0.614
Total cholesterol (mmol/L)	0.01	0.940	−0.02	0.892
HDL-C (mmol/L)	−0.18	0.194	−0.01	0.928
LDL-C (mmol/L)	0.09	0.488	−0.01	0.962
Non-HDL-C (mmol/L)	0.09	0.541	−0.01	0.939
hsCRP (mg/L)	0.23	0.101	0.01	0.919
	**AGE/sRAGE Ratio (AU/pg)**			
	**Before ALA**		**After ALA**	
	**r**	***p***	**r**	***p***
Age (years)	0.07	0.596	0.07	0.596
BMI (kg/m^2)	0.08	0.547	−0.07	0.653
Glucose (mmol/L)	−0.17	0.227	0.17	0.272
HbA1C (%)	−0.07	0.619	−0.18	0.253
Creatinine (µmol/L)	−0.09	0.497	−0.03	0.825
Uric Acid (µmol/L)	0.08	0.566	0.23	0.144
Total cholesterol (mmol/L)	0.06	0.642	0.15	0.336
HDL-C (mmol/L)	0.31	0.273	0.30	0.060
LDL-C (mmol/L)	−0.01	0.967	0.07	0.663
Non-HDL-C (mmol/L)	−0.06	0.674	0.02	0.891
hsCRP (mg/L)	−0.26	0.063	−0.03	0.840

Abbreviations: AGE, advance glycation end product; ALA, alpha-lipoic acid; BMI, body mass index; HbA1C; HDL-C, high-density lipoprotein cholesterol; hsCRP, high-sensitivity C-reactive protein; LDL-C, low-density lipoprotein cholesterol; sRAGE, soluble receptor of advance glycation end product.

Variables with non-normal distribution were logarithmized before correlation analyses.
